# Assessment of Cognitive Emotion Regulation in Gambling Disorder: A Systematic Review of the Literature

**DOI:** 10.3390/clinpract16030056

**Published:** 2026-03-05

**Authors:** Ioana Ioniță, Mădălina Iuliana Mușat, Bogdan Cătălin, Adela Magdalena Ciobanu

**Affiliations:** 1 “Carol Davila” Doctoral School, Faculty of Medicine, University of Medicine and Pharmacy, 050474 Bucharest, Romania; ioana_ionita@ymail.com; 2 Experimental Research Center for Normal and Pathological Aging, University of Medicine and Pharmacy of Craiova, 2 Petru Rareș Street, 200349 Craiova, Romania; 3 Department of Scientific Research Methodology, University of Medicine and Pharmacy of Craiova, 2 Petru Rareș Street, 200349 Craiova, Romania; 4 Department of Physiology, University of Medicine and Pharmacy of Craiova, 2 Petru Rareș Street, 200349 Craiova, Romania; 5 Department of Psychiatry, “Prof. Dr. Alexandru Obregia” Clinical Hospital of Psychiatry, 041914 Bucharest, Romania; adela.ciobanu@yahoo.com; 6 Neuroscience Department, Discipline of Psychiatry, Faculty of Medicine, “Carol Davila” University of Medicine and Pharmacy, 020021 Bucharest, Romania

**Keywords:** CERQ, cognitive emotion regulation, emotion regulation, ERQ, gambling disorder, impulsivity

## Abstract

**Background/Objectives:** Gambling disorder (GD) is a behavioral addiction characterized by persistent and repetitive gambling behaviors that cause significant psychological distress and functional impairment. Increasing evidence indicates that difficulties in emotion regulation are a key factor in the development and persistence of GD. This systematic review aimed to summarize and critically evaluate the existing literature on the relationship between emotion regulation strategies and gambling disorder, with a specific focus on studies using the Emotion Regulation Questionnaire (ERQ) and the Cognitive Emotion Regulation Questionnaire (CERQ). **Methods:** The review was conducted according to the Preferred Reporting Items for Systematic Reviews and Meta-Analyses (PRISMA 2020) guidelines. Systematic searches were performed in PubMed and Scopus databases for studies published between 25 October 2015 and 25 October 2025. The methodological quality and risk of bias of the included studies were evaluated using the Joanna Briggs Institute (JBI) Critical Appraisal Checklist and JBI Checklist for Randomized Controlled Trials. Data extraction and synthesis were performed manually by two independent reviewers. Eligible studies included adult participants (≥18 years) diagnosed with gambling disorder or pathological gambling and using the ERQ or CERQ to assess emotion regulation. **Results:** Nine studies met the inclusion criteria, comprising a total of 607 patients with GD. Across studies, individuals with GD consistently showed reduced cognitive reappraisal, greater expressive suppression, and higher use of maladaptive cognitive strategies such as rumination, catastrophizing, and self-blame. All studies identified impulsivity, emotion dysregulation, alexithymia, or gambling-related cognitive distortions as significant predictors of gambling severity. Neuroimaging evidence from one study further revealed altered activation of frontal regions during negative emotion regulation. **Conclusions:** This review highlights the central role of emotion regulation in GD. However, the limited available ERQ/CERQ studies in GD were mostly cross-sectional, limiting causal inferences. Second, samples were predominantly male, reducing generalizability to women. Finally, only one study used neurobiological measures, hindering integration of self-report and neural data. These findings emphasize the importance of integrating emotion regulation-based interventions within therapeutic programs for gambling disorder, with ERQ and CERQ being useful tools to assess the pathology.

## 1. Introduction

Gambling disorder (GD) is recognized as a behavioral addiction characterized by persistent and recurrent maladaptive gambling behaviors that lead to significant distress or impairment in personal, social, or occupational functioning [1,2]. In the Diagnostic and Statistical Manual of Mental Disorders, Fifth Edition (DSM-5), GD is classified alongside substance-related disorders, reflecting shared features such as craving, loss of control, and the persistence of the behavior despite adverse consequences. These similarities have prompted growing interest in exploring the underlying psychological and neurocognitive mechanisms that contribute to the onset and maintenance of GD [2,3].

GD represents a condition associated with substantial clinical burden and functional impairment [4,5]. Individuals with GD frequently experience severe financial difficulties, interpersonal conflicts, occupational instability, and legal consequences, all of which significantly compromise quality of life [6,7]. Psychiatric comorbidity is highly prevalent, with elevated rates of major depressive disorder, anxiety disorders, and substance use disorders and increased suicidal ideation and attempts [4,8]. From a psychopathological perspective, GD is maintained by a complex interaction between impulsivity, maladaptive cognitive distortions, emotional dysregulation, and reinforcement learning mechanisms [9]. Among the various psychological factors implicated in GD, emotion regulation has emerged as a key construct. Emotion regulation refers to the processes by which individuals influence the intensity, duration, and expression of their emotional responses [10,11,12]. Deficits in emotion regulation are thought to play a central role in the development of addictive behaviors, serving both as a predisposing vulnerability and a maintaining factor in maladaptive gambling [13,14]. In line with vulnerability and maintenance models of addictive behaviors, trauma and affect dysregulation mediated the pathway from insecure attachment to dissociation, while impulsivity, compulsivity, and obsessiveness mediated the association between dissociation and affect dysregulation [15,16]. Individuals with GD frequently report using gambling as a strategy to cope with negative emotional states, to escape stress, or to enhance positive affect, thereby reinforcing the addictive cycle [11,12,13,14].

Two validated self-report instruments are commonly employed to assess emotion regulation: the Emotion Regulation Questionnaire (ERQ) [17,18,19] and the Cognitive Emotion Regulation Questionnaire (CERQ) [20,21,22,23]. The ERQ focuses on two key strategies—cognitive reappraisal (an adaptive form of emotion regulation) and expressive suppression (a maladaptive strategy associated with poorer psychological outcomes) [17,18,19]. The CERQ, on the other hand, provides a broader assessment of nine cognitive strategies that individuals use in response to emotionally activating events, distinguishing between adaptive and maladaptive regulation styles [20,21,22,23]. Understanding how these emotion regulation patterns relate to gambling behavior offers valuable insights into the cognitive–affective mechanisms underlying GD. However, the existing literature remains fragmented and marked by several important gaps. In particular, there is a lack of systematic integration of findings derived specifically from ERQ and CERQ measures, limited clarity regarding the consistency of emotion regulation profiles associated with GD, and insufficient evidence linking these strategies to gambling severity across adult populations. Although multiple instruments exist to assess emotion regulation, the present review was designed as an instrument-specific synthesis focusing on the ERQ and CERQ. These tools were selected due to their strong theoretical grounding, widespread international use, and clear differentiation between adaptive and maladaptive cognitive regulation strategies. Focusing on these two instruments allowed for improved conceptual consistency and greater comparability across studies, thereby strengthening the interpretability of findings.

Therefore, the present study aims to systematically review the existing literature on emotion regulation and GD, focusing on studies employing the ERQ and CERQ instruments. Specifically, the review seeks to (1) summarize the main findings and methodological characteristics of published studies, (2) identify common emotion regulation patterns among individuals with GD, (3) highlight key psychological predictors associated with gambling severity. By integrating current evidence, this review contributes to a better understanding of the emotional and cognitive mechanisms implicated in GD and may inform future preventive and therapeutic strategies and aims to address the following research question: How are emotion regulation strategies, as measured by the ERQ and CERQ, associated with GD and gambling severity in adults and what common emotion regulation patterns are observed among individuals with GD?

## 2. Methods

### 2.1. Design and Search Strategy

A systematic review was carried out in accordance with the Preferred Reporting Items for Systematic Reviews and Meta-Analyses (PRISMA) guidelines [24]. Due to the heterogeneity of study designs and outcomes across the included studies, performing a quantitative meta-analysis would not have been appropriate, as pooled effect estimates would have been potentially misleading. The PRISMA 2020 checklist (Supplementary Materials File S1) corresponds to the official reporting guideline template and is provided in its standardized format [24]. The review protocol was registered in Open Science Framework available at Registration https://doi.org/10.17605/OSF.IO/VX52J. The electronic databases PubMed and Scopus were searched for relevant studies published between 25 October 2015 and 25 October 2025, with the final search conducted on 25 October 2025. The selected time window (2015–2025) was chosen to reflect the period during which research specifically employing the ERQ and CERQ in GD populations began to emerge more consistently, following the DSM-5 reclassification of GD from the Impulse Control Disorders Not Elsewhere Classified chapter to the newly expanded Substance-Related and Addictive Disorders section [25]. PubMed and Scopus were selected due to their broad and complementary coverage of biomedical, psychiatric, and behavioral science literature relevant to GD and cognitive emotion regulation.

The search strategy included a combination of terms: “cognitive emotion regulation questionnaire” or “cognitive emotion regulation” or “CERQ” AND “gambling disorder” or “pathological gambling” or “gambling”. All records retrieved using these combinations of terms were subsequently examined manually to assess their eligibility based on the predefined inclusion and exclusion criteria.

### 2.2. Inclusion/Exclusion Criteria

The selection of studies was guided by clearly defined inclusion and exclusion criteria to ensure methodological rigor (Table 1).

### 2.3. Selection Process, Data Extraction and Synthesis

The study selection process was conducted in accordance with the PRISMA 2020 guidelines [24]. Data extraction and synthesis were performed independently by two reviewers, and discrepancies arising at any stage of the review were addressed through consensus discussions, with arbitration by a third reviewer when required. Supplementary Materials (File S2) provides a detailed description of the variables included in the data extraction process.

**Figure 1:**
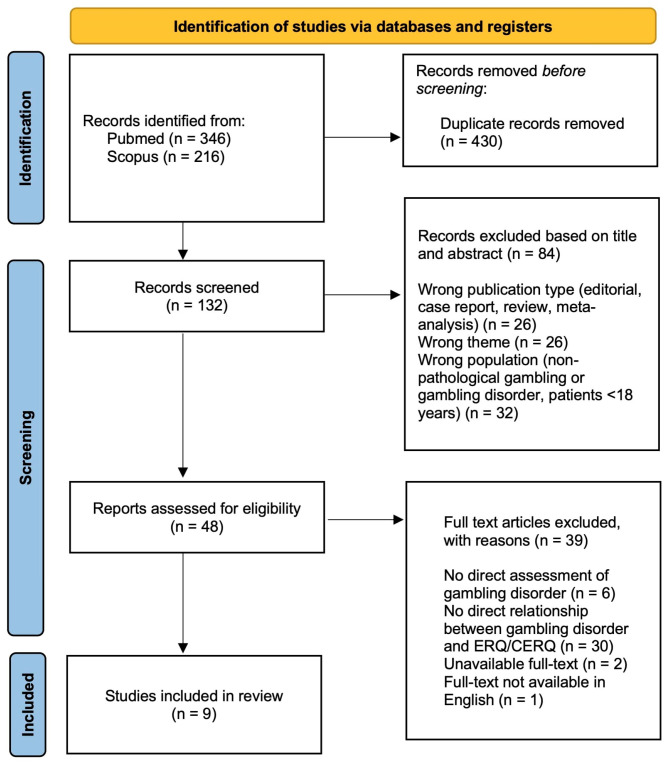
PRISMA flow diagram of the literature screening and selection process.

The process included the following stages:

• Identification: An initial search was performed in the databases using the specified combinations of search terms. All retrieved records were exported into a bibliographic file for further analysis.
• Removal of duplicates: Duplicate articles were manually identified and removed.
• Screening (title and abstract): Titles and abstracts were independently reviewed to assess their relevance. Studies that did not meet the inclusion criteria were excluded at this stage.
• Eligibility: The full texts of the selected articles were read and analyzed to confirm their eligibility.
• Inclusion: Studies that met all inclusion criteria were incorporated into the final analysis. The total of 9 studies were included as presented in the PRISMA flow diagram (Figure 1).

### 2.4. Risk of Bias and Quality Assessment

The methodological quality and risk of bias of the included studies were evaluated using the Joanna Briggs Institute (JBI) Critical Appraisal Checklist for Analytical Cross-Sectional Studies and JBI Checklist for Randomized Controlled Trials [26], by two reviewers to minimize bias, with disagreements resolved by consensus.

JBI Critical Appraisal Checklist for Analytical Cross-Sectional Studies assessed 8 key domains: (Q1) clarity of inclusion criteria, (Q2) detailed description of study subjects and setting, (Q3) validity and reliability of exposure measurement, (Q4) use of objective and standard criteria for condition measurement, (Q5) identification of confounding factors, (Q6) strategies to manage confounding factors, (Q7) validity and reliability of outcome measurement, (Q8) appropriateness of statistical analysis.

JBI Checklist for Randomized Controlled Trials assessed 13 key domains: (Q1) use of true randomization for assignment of participants to treatment groups, (Q2) concealment of allocation to treatment groups, (Q3) similarity of treatment groups at baseline, (Q4) blinding of participants to treatment assignment, (Q5) blinding of those delivering treatment to treatment assignment, (Q6) blinding of outcome assessors to treatment assignment, (Q7) identical treatment of groups other than the intervention of interest, (Q8) completeness of follow-up and appropriate description and analysis of differences between groups, (Q9) analysis of participants in the groups to which they were randomized, (Q10) consistency of outcome measurement across treatment groups, (Q11) reliability of outcome measurement, (Q12) appropriateness of statistical analysis, (Q13) appropriateness of trial design and accounting for any deviations from the standard RCT design.

## 3. Results

### 3.1. Study Characteristics

The main characteristics of the studies included in the systematic review are summarized in Table 2. Most studies employed a cross-sectional or case–control design, with one randomized controlled trial evaluating a cognitive behavioral therapy (CBT) intervention combined with a serious gaming approach, comprising a total of 607 patients with GD.

Sample sizes varied considerably, including controls, ranging from 44 to 246 participants, and most samples were predominantly male, reflecting the gender imbalance commonly observed in GD research. Mean participant ages ranged from 25.7 to 47.7 years, depending on the study population and design.

### 3.2. Quality Assessment of the Studies Included (Risk of Bias)

**Figure 2:**
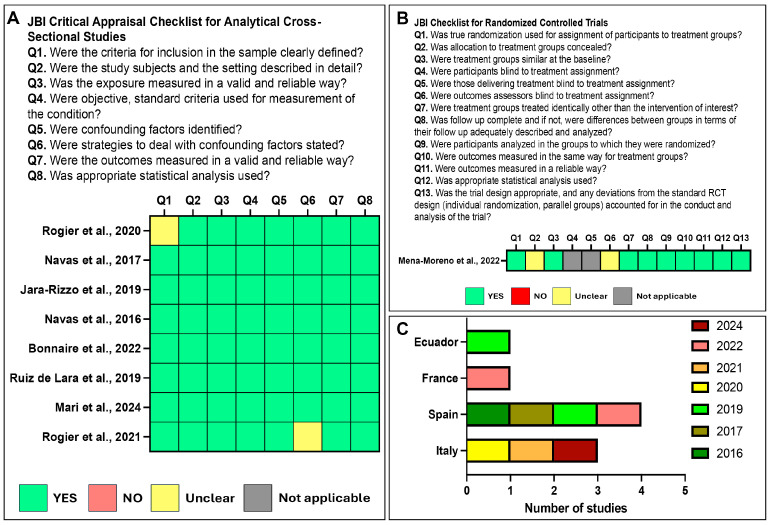
Assessment of the included studies. (**A**) Risk of bias summary for each study based on the Joanna Briggs Institute (JBI) Critical Appraisal Checklist for Analytical Cross-Sectional Studies, covering eight methodological domains (Q1–Q8). Green squares indicate “Yes” (criterion met), yellow “Unclear,” red “No” (criterion not met), and grey “Not applicable” [9,27,28,29,30,31,32,34]. (**B**) Risk of bias based on JBI Checklist for Randomized Controlled Trials, covering 13 methodological domains (Q1–Q13 [33]. (**C**) Distribution of the included studies by country and year of publication.

Overall, the studies demonstrated good methodological quality. In JBI Critical Appraisal Checklist for Cross-Sectional studies, most items were rated as “Yes,” indicating a low risk of bias across domains (Figure 2A). Only 2 studies showed “Unclear” ratings for the description of inclusion criteria or confounding factor management. Using the JBI Checklist for Randomized Controlled Trials, 9 of 13 domains were rated as “Yes”, two as “Not applicable” (participant and provider blinding), and two as “Unclear” (allocation concealment and outcome assessor blinding) (Figure 2B). No study was assessed as having a high risk of bias.

The geographical distribution of the included studies is also presented in Figure 2C, indicating that research originated primarily from Spain and Italy, with additional contributions from France and Ecuador over the period 2016–2024.

### 3.3. Overview of CERQ/ERQ Instruments, Associated Measures, and Key Findings

Across the included studies, individuals with GD consistently showed altered emotion regulation profiles compared with controls. Using the ERQ, several studies reported lower cognitive reappraisal and higher expressive suppression in GD. For instance, Rogier et al. (2020) [27] found lower reappraisal in gamblers than controls (28.11 ± 7.24 vs. 31.18 ± 6.22, *p* < 0.001) and higher suppression (16.24 ± 5.75 vs. 14.07 ± 5.62, *p* = 0.045). Similar results were observed by Rogier et al. (2021) [34], where reappraisal was negatively associated with gambling severity (r = −0.35, *p* < 0.001), whereas suppression showed a positive association (r = 0.27, *p* = 0.008).

Findings from CERQ-based studies indicated greater use of maladaptive strategies among GD patients, particularly self-blame and catastrophizing (Navas et al., 2016) [30]. Notably, some strategies usually considered adaptive, such as putting into perspective, were paradoxically linked to greater gambling severity and stronger gambling-related cognitive distortions. Ruiz de Lara et al. (2019) further showed that reappraisal and rumination predicted gambling-related cognitive biases [32].

Emotion regulation strategies were also related to behavioral indicators of gambling. In Mari et al. (2024) [9], risky betting was negatively correlated with cognitive reappraisal (r = −0.43, *p* < 0.01) and positively correlated with emotion dysregulation (r = 0.44, *p* < 0.01). In the only randomized controlled trial, Mena-Moreno et al. (2022) [33] reported a significant reduction in expressive suppression following the intervention, accompanied by improved clinical outcomes (Table 3).

## 4. Discussion

### 4.1. Summary of Evidence

This systematic review focused on how emotion regulation strategies, as assessed by the ERQ and CERQ, are related to GD and gambling severity in adults, and on identifying the common emotion regulation patterns observed among individuals with GD. The findings of this systematic review highlight a consistent pattern of emotion regulation impairments among individuals with GD, underscoring the central role of both maladaptive cognitive strategies and reduced use of adaptive regulation in the maintenance of the disorder.

Across the nine included studies, a consistent pattern emerged demonstrating that adults with GD show marked impairments in emotion regulation, as measured by both ERQ and CERQ. Most studies reported lower cognitive reappraisal and higher expressive suppression among individuals with GD compared with healthy or recreational gambling controls, supporting previous meta-analytic findings linking GD with pervasive emotion dysregulation [12].

Maladaptive cognitive regulation strategies, particularly self-blame, catastrophizing, rumination, and heightened reliance on suppression, were associated with increased gambling severity. CERQ-based evidence indicated not only elevated maladaptive strategies but also paradoxical associations between some typically “adaptive” strategies (e.g., putting into perspective, refocusing on planning) and greater cognitive distortions or severity, suggesting that individuals with GD may use these strategies in rigid or context-inappropriate ways. This aligns with prior work highlighting dysfunctional emotion regulation profiles across behavioral addictions [10,11]. The associations observed between emotion regulation strategies and GD may be explained by several underlying psychological and neurocognitive mechanisms. Maladaptive strategies such as expressive suppression, rumination, and catastrophizing are known to increase emotional arousal while reducing emotional clarity, thereby intensifying negative affect and impairing adaptive coping. In this context, gambling may function as an avoidance-based strategy aimed at temporarily escaping or numbing aversive emotional states. Conversely, reduced use of cognitive reappraisal may limit the individual’s ability to reinterpret gambling-related losses or distressing experiences in a flexible and adaptive manner, increasing reliance on impulsive, short-term reward-seeking behaviors.

An additional robust predictor across studies was impulsivity, particularly negative and positive urgency, which denotes rash action in the context of intense affect. These findings are consistent with broader research showing that urgency-related impulsivity is strongly associated with addictive behaviors and heightened reactivity to emotional states [13,14,35,36]. GD often sets in motion a cascade of psychological and behavioral vulnerabilities, whereby the emotional volatility it generates increases reliance on alcohol as a means of temporary relief [37,38,39,40], a dependence that gradually deepens underlying depression and anxiety [41,42], which then further fuel the urge to gamble [43,44], creating a self-perpetuating cycle in which each component intensifies and sustains the others [45]. Moreover, urgency-related impulsivity may further amplify this process by promoting rash decision-making under emotional pressure, thereby strengthening the link between emotional dysregulation and gambling behavior.

Neuroimaging evidence [30] further supported the presence of altered frontal activation patterns during cognitive reappraisal, indicating that GD is characterized not only by self-reported regulation difficulties but also by measurable neurobiological dysregulation. Neuroimaging in psychiatry has advanced substantially in recent years, yet its clinical use remains far behind its scientific potential, even though growing evidence shows that brain-based markers can meaningfully inform diagnosis, prognosis and treatment response. Despite this robust evidence base, neuroimaging is still used mainly for exclusion of organic pathology rather than to characterize disease mechanisms or personalize interventions [46]. By incorporating neuroimaging more routinely into psychiatric evaluation and research, clinicians could better identify subtypes, tailor treatments according to neurocognitive profiles and ultimately improve long-term outcomes [47].

Overall, results indicate that maladaptive emotion regulation is not simply a correlate but a core maintaining mechanism of GD, supporting the integration of emotion regulation-focused interventions (e.g., CBT with ER training, serious gaming approaches) [48,49,50,51,52,53]. Recent clinical trials demonstrating reductions in expressive suppression and relapse rates following such interventions provide preliminary evidence for their therapeutic value [33,50].

From a clinical perspective, the present findings suggest that the ERQ and CERQ may represent useful tools for both assessment and treatment planning in GD. Elevated expressive suppression and reduced cognitive reappraisal, as well as increased use of maladaptive cognitive strategies (e.g., self-blame, rumination, and catastrophizing), could serve as clinically relevant markers of emotional vulnerability. Routine assessment of these strategies may help clinicians identify patients at higher risk of emotion-driven gambling and relapse. In terms of intervention, treatment approaches may benefit from explicitly targeting emotion regulation processes, for example by promoting cognitive reappraisal skills, reducing reliance on expressive suppression, and addressing maladaptive cognitive regulation patterns. These targets are consistent with cognitive–behavioral and emotion-focused interventions and may contribute to improved emotional coping, reduced impulsive decision-making under distress, and greater resistance to gambling urges. Beyond their descriptive value, ERQ and CERQ findings may be integrated into a structured clinical workflow in GD. At the assessment stage, these instruments may serve as screening tools to identify dominant maladaptive regulation profiles. During case formulation, emotion regulation patterns may be mapped onto specific gambling triggers, thereby clarifying the functional role of gambling behavior. In treatment planning, such differentiation may guide the selection of targeted intervention components, such as behavioral activation and affect exposure for suppression-dominant profiles [54], or cognitive restructuring and metacognitive techniques for rumination/catastrophizing profiles [55,56,57]. In relapse prevention, repeated ERQ/CERQ assessment may help detect early shifts toward maladaptive regulation patterns before behavioral relapse becomes clinically evident [58]. This mechanism-oriented integration may enhance personalization and improve long-term treatment stability in GD.

### 4.2. Strengths and Limitations

This systematic review has several notable strengths. First, it is, to our knowledge, the first review to focus specifically on emotion regulation in GD using two well-established and theoretically grounded instruments, the ERQ and the CERQ. By restricting inclusion to studies employing these measures, the review ensures conceptual consistency. Second, the review followed a registered protocol and was conducted in accordance with PRISMA 2020 guidelines, with independent screening, data extraction, and quality appraisal performed by two reviewers, thereby reducing selection and assessment bias. Third, only studies including adult participants formally diagnosed with GD using standardized diagnostic criteria or recruited in a clinical setting were considered. Although this approach resulted in a limited number of eligible studies, it strengthened the clinical relevance and internal validity of the synthesized evidence.

This review has several limitations that should be considered when interpreting its findings. First, the literature search was limited to PubMed and Scopus. Although these databases offer broad multidisciplinary coverage and are widely used in biomedical and psychological research, it is possible that relevant studies indexed exclusively in other databases were not identified. Second, only English-language full-text articles were included, which may have introduced language bias and resulted in the exclusion of potentially relevant studies published in other languages. These restrictions may have influenced the completeness of the evidence base and should be considered when interpreting the findings. Future reviews could benefit from including additional databases and non-English literature. The restriction to the 2015–2025 interval may have excluded earlier studies examining broader emotion regulation constructs using alternative measures. However, this decision was made to ensure conceptual consistency.

An additional limitation of this review concerns the geographical representation of the included studies. The present review employed highly stringent inclusion criteria, restricting eligibility to studies involving adult participants formally diagnosed with GD in a clinical context or according to validated diagnostic criteria. As a result, studies conducted in certain regions were excluded because they primarily examined community samples, recreational or occasional gamblers, or individuals without a confirmed diagnosis of GD. While this approach enhances the clinical specificity and internal validity of the findings, it may limit the generalizability of the results across different cultural and regulatory gambling environments.

Another source of heterogeneity concerns the diagnostic criteria used across the included studies. Most investigations relied on standardized clinical diagnostic systems (DSM-IV or DSM-5), reflecting the period in which the data were collected. However, one study [27] used only the SOGS as a diagnostic proxy rather than a formal DSM-based diagnosis. Although SOGS is a widely used and validated screening instrument, it does not fully correspond to current diagnostic criteria for GD and may reflect a broader or partially different clinical profile.

The number of available studies using ERQ or CERQ in GD is limited, and most included studies were cross-sectional. The present findings should be interpreted in light of the predominance of cross-sectional study designs among the included articles. The available evidence does not allow conclusions regarding the directionality or causality of these relationships. It remains unclear whether deficits in emotion regulation represent a vulnerability factor for the development of GD or whether they emerge as a consequence of prolonged gambling behavior. Therefore, the identified emotion regulation patterns should be interpreted as correlates of GD rather than as causal mechanisms.

Although the included studies were conducted in predominantly male samples, emotion regulation processes may operate differently in men and women. Women with GD have been shown in other areas of addiction research to exhibit higher levels of negative affect, emotion-focused coping, and internalizing symptoms, whereas men more frequently display externalizing behaviors and impulsivity-driven pathways. These differences suggest that maladaptive strategies such as rumination, self-blame, or catastrophizing may play a more prominent role in gambling behavior among women, while impulsivity-related strategies and expressive suppression may be more central among men. However, due to the limited representation of female participants in the reviewed studies, these potential gender-specific pathways remain largely speculative.

In addition, demographic heterogeneity was evident, with samples differing markedly in age ranges and gender distribution and being predominantly male in most studies. Sample sizes varied widely, and several studies relied on relatively small clinical groups, which may have reduced statistical power and increased the likelihood of unstable effect estimates. Together, these sources of heterogeneity may have affected the comparability of results across studies and underscore the need for caution when synthesizing findings and for more methodologically consistent and adequately powered research in future investigations.

Additionally, only one study employed neurobiological measures, limiting the integration of self-report data with objective neural correlates. Finally, intervention-based evidence remains scarce, with only one randomized controlled trial included, underscoring the need for more rigorous clinical research evaluating emotion regulation as a therapeutic target.

### 4.3. Future Directions

Future research should aim to address the identified gaps by employing longitudinal designs that can clarify causal mechanisms linking cognitive emotion regulation deficits to the onset and maintenance of GD. More studies incorporating gender-balanced samples are essential to understand potential sex-specific mechanisms and to tailor interventions accordingly. Advancing the field will require the adoption of standardized outcome measures for both emotion regulation and gambling severity, facilitating more direct comparisons across studies and improving the synthesis of findings. Further investigations would benefit from greater methodological standardization and consistent reporting of statistical metrics. Such uniformity would facilitate more precise cross-study comparisons and allow for meaningful quantitative syntheses and meta-analytic integration in this field.

In addition, integrating multimodal assessment approaches, including neuroimaging, psychophysiological markers, and ecological momentary assessment, would significantly advance understanding of how cognitive emotion regulation processes operate in real time. Further future investigations should prioritize longitudinal designs and randomized clinical trials with larger and more culturally diverse samples in order to clarify the directionality and causal mechanisms linking emotion regulation strategies to GD. In addition, greater methodological consistency in the assessment of emotion regulation and gambling-related outcomes would facilitate quantitative synthesis and more precise estimation of effect sizes. Addressing these gaps is essential for advancing the field beyond descriptive associations toward clinically actionable models of vulnerability and intervention.

## 5. Conclusions

This systematic review indicates that individuals with GD consistently exhibit deficits in emotion regulation, including reduced cognitive reappraisal, heightened expressive suppression, and increased reliance on maladaptive cognitive strategies. These patterns are closely intertwined with impulsivity, emotion-driven decision-making, and gambling-related cognitive distortions. Together, they highlight emotion regulation impairments as a central psychological mechanism in GD. Addressing these deficits, through assessment using ERQ and CERQ and through targeted therapeutic approaches, may enhance treatment outcomes and reduce relapse risk. Because most included studies employed cross-sectional designs, the conclusions of this review are necessarily limited to associations rather than causal relationships. Longitudinal and experimental studies are required to determine whether emotion regulation deficits precede the onset of GD or result from its progression. Continued research is needed to develop and refine cognitive emotion regulation-based interventions and to expand the evidence base with more diverse and methodologically robust studies.

## Figures and Tables

**Table 1:** Inclusion and exclusion criteria.

Criteria	Inclusion Criteria	Exclusion Criteria
Study Type	Original research articles.	Reviews, meta-analyses, case reports, editorials.
Population	Adult participants (≥18 years) diagnosed with or evaluated for gambling disorder or pathological gambling.	Studies involving adolescents, general populations without gambling disorder, or non-human subjects.
Focus/Variables	Studies examining the relationship between gambling disorder and cognitive/emotion regulation, particularly using the ERQ or CERQ scales.	Studies without a direct measure of cognitive/emotion regulation or gam-bling disorder.
Publication Language	Articles published in English with full-text available.	Articles with no full-text available or not published in English.
Publication Date	Published between 25 October 2015 and 25 October 2025.	Published outside the specified date range.

**Table 2:** Characteristics of the Included Studies.

Study Identification	Country	Study Design	Sample (n)	M/F Distribution	Age (Mean ± SD) Years	Diagnostic Criteria	Type of Gambling	Study Limitation
Rogier et al., 2020 [27]	Italy	Cross-sectional study	79 (clinical group) + 101 (control group)	81% male (addicted gamblers), 79% male (controls)	47.73 ± 13.50 (gamblers); 46.88 ± 10.09 (controls)	SOGS	Various gambling types (unspecified)	-Gender imbalance; -Reliance on self-report measures; -Gambling type not considered; -Limited generalizability due to cultural factors.
Navas et al., 2017 [28]	Spain	Cross-sectional case–control study	Study 1: 41 (GD) + 45 (controls); Study 2: 17 (GD) + 21 (controls)	Study 1: 100% male; Study 2: GD patients 16 M/1 F; controls 20 M/1 F	Study 1: GD patients 35.22 ± 11.16; controls 33.22 ± 8.18 Study 2: GD patients 32.94 ± 7.77; controls 31.00 ± 4.60	DSM-IV	Strategic (card games, sport bets) and passive gambling modalities (slot machine, lotteries)	-The emotions triggered by the cognitive reappraisal task were less intense than those experienced in daily life; -Small sample size limiting statistical power, particularly for neuroimaging analyses.
Jara-Rizzo et al., 2019 [29]	Ecuador	Cross-sectional study	197 (patients = 27, community gamblers = 170)	Patients: 26% females; Community: 39% females	Patients: 25.74 ± 8.34; Community: 34.36 ± 13.73	DSM-IV and SOGS	Various gambling types (unspecified)	-Gambling type not considered; -Subtle effects and measurement error, partly due to the use of proxy measures for key constructs.
Navas et al., 2016 [30]	Spain	Cross-sectional case–control study	41 (GD) + 45 (controls)	100% males	GD patients: 35.22 ± 11.16; controls: 33.22 ± 8.18	DSM-IV	Various gambling types (unspecified)	-Sample composed only of males, limiting generalizability to females; -Gambling type not considered; -Limited sample size, reduced statistical power.
Bonnaire et al., 2022 [31]	France	Cross-sectional study	59 (clinical group) + 107 (non-problematic community gamblers)	Clinical: 16.9% females and 83.1% males; Non-clinical: 38.3% females and 61.7% males	Clinical: 41.37 ± 11.42; Non-clinical: 38.74 ± 9.42	DSM-5	Mixed gambling modalities: chance-based games (lottery, scratch cards, slot machine) and skills-based games (poker, sport betting, horse race betting)	-Relatively small sample size in clinical group, reducing statistical power and possibly obscuring relevant factors; -Limited number of instruments per pathway, restricting comprehensive assessment of emotion regulation and impulsivity constructs.
Ruiz de Lara et al., 2019 [32]	Spain	Cross-sectional study	30 (GD − DSM-5) + 20 (community gamblers − SOGS) + 196 (non-GD community gamblers)	82 females, 164 males	33.14 ± 13.88	DSM-5 and SOGS	Type I (cards, casino games, skill games, sports bets) and Type II (lotteries, pools, bingo, slot machines)	-Small to medium effect sizes, partly due to measurement error and use of proxy measures for key constructs; -Limited sample size for subgroup comparisons (e.g., gambling modality or gambling motives).
Mari et al., 2024 [9]	Italy	Cross-sectional study	24 (GD), 20 (Substance-Dependent Gambling − SDG), 20 (healthy controls − HC)	100% male	GD: 35.89 ± 11.78 SDG: 35.10 ± 11.88 HC: 34.95 ± 12.80	DSM-5	Various gambling types (unspecified)	-Gambling type not considered; -Relatively small sample size, limiting generalizability of the findings. -Only male participants were included, preventing extension of results to female gamblers.
Mena-Moreno et al., 2022 [33]	Spain	Randomized controlled study with CBT intervention + serious game	40 (experimental group) + 67 (control group)	95.2% male	40.2 ± 14.7	DSM-5	Various gambling types (unspecified)	-Gambling type not considered; -Predominantly male sample (only five women); -Trait impulsivity and emotion regulation (UPPS-P, DERS, ERQ) could not be compared between experimental and control groups; -Limited number of intervention sessions.
Rogier et al., 2021 [34]	Italy	Cross-sectional study	79 (clinical group), 70 (control group)	Clinical: 84.81% males; Control: 82.86% males	Clinical: 46.48 ± 13.59; Control: 46.14 ± 12.12	DSM-5	Slot machines/video Lottery, sports betting, mixed online/offline gambling	-Relatively small sample size, limiting generalizability and subgroup analyses; -The Italian translation of the DES-II used in this study has not been validated.

Abbreviations: Gambling Disorder (GD), South Oaks Gambling Screen (SOGS), Diagnostic and Statistical Manual of Mental Disorders (DSM), Cognitive behavioral therapy (CBT).

**Table 3:** Instruments, Variables and Main Findings.

Study Identification	CERQ/ERQ Versions	CERQ/ERQ Versions Description	Other Measures	Main Findings (GD Compared to Controls)	Key Predictors of GD Severity
Rogier et al., 2020 [27]	ERQ	10-item self-report questionnaire with 2 scores: Cognitive Reappraisal and Expressive Suppression	Personality Inventory for DSM-5 (PID-5), DERS, SOGS	-Gamblers showed lower cognitive reappraisal and higher expressive suppression vs. controls, and higher scores in DERS and PID-5 domains; -ERQ: Reappraisal: Gamblers = 28.11 ± 7.24, Controls = 31.18 ± 6.22, F = 13.59, *p* < 0.001; Suppression: Gamblers = 16.24 ± 5.75, Controls = 14.07 ± 5.62, F = 4.08, *p* = 0.045.	-Impulsivity; -Suspiciousness; -Perseveration; -Emotion Dysregulation.
Navas et al., 2017 [28]	ERQ—Spanish version	10-item ERQ: 6 items for Cognitive Reappraisal, 4 items for Expressive Suppression	SOGS, UPPS-P Impulsive Behavior Scale (focus on Negative Urgency), Multicage CAD-4, IQ (WAIS-IV or KBIT), functional magnetic resonance imaging (fMRI) Cognitive Reappraisal Task (Study 2)	-GD patients showed higher emotional suppression compared to controls (GD = 17.37 ± 5.38, HC = 14.93 ± 5.21, F (1,84) = 4.525, *p* = 0.036); -No differences in reappraisal between groups (GD = 29.07 ± 5.37, HC = 30.05 ± 7.11, F (1,84) = 0.503, *p* = 0.480); -GD patients showed hyperactivation of left premotor and middle frontal gyri during regulation of negative emotions; -Negative urgency correlated positively with emotional suppression and middle frontal gyrus activation during negative emotion regulation.	-Negative urgency significantly associated with emotional suppression and with left middle frontal activation; -The use of emotional suppression and increased activation of the middle frontal gyrus during negative emotion regulation are associated with impulsivity driven by negative emotions.
Jara-Rizzo et al., 2019 [29]	ERQ—Spanish version	10-item ERQ: 6 items for Cognitive Reappraisal, 4 items for Expressive Suppression	SOGS, GRCS, UPPS-P Impulsive Behavior Scale, MultiCAGE CAD-4	-Reappraisal was positively associated with gambling-related cognitive distortions (GRCS) (R^2^ = 0.079, *p* < 0.001); -Suppression was associated with higher levels of negative urgency (emotion-driven impulsivity) (z = 2.132, *p* = 0.033); -Positive urgency and sensation seeking were associated with gambling-related cognitive biases (illusion of control β = 0.188, *p* = 0.011; predictive control β = 0.188, *p* = 0.018; interpretative bias β = 0.140, *p* = 0.043).	-Positive urgency and sensation seeking were significant predictors of gambling-related cognitive distortions, which are linked to GD severity; -Negative urgency was associated with maladaptive emotion regulation (suppression).
Navas et al., 2016 [30]	CERQ—Spanish version	27 5-point Likert scale items assessing 9 strategies: Self-blame, Other-blame, Rumination, Catastrophizing, Putting into perspective, Positive refocusing, Positive reappraisal, Acceptance, Refocus on planning	SOGS, GRCS, MultiCAGE CAD-4	-GD patients showed higher scores than controls across all GRCS dimensions; -GD patients used self-blame, catastrophizing, and positive refocusing more frequently than controls (Self-blame: GDPs = 11.05 ± 3.09, HCs = 7.20 ± 2.54, F = 40.14, *p* < 0.01, η^2^ = 0.32; Catastrophizing: GDPs = 8.10 ± 2.74, HCs = 6.02 ± 1.91, F = 16.85, *p* = 0.01, η^2^ = 0.17; Positive refocusing: GDPs = 9.27 ± 2.26, HCs = 7.96 ± 2.87, F = 5.49, *p* = 0.02, η^2^ = 0.06); -Some “adaptive” emotion regulation strategies (e.g., putting into perspective, refocusing on planning) were paradoxically associated with greater gambling severity and cognitive distortions; -Putting into perspective: GDPs = 10.61 ± 2.82, HCs = 9.36 ± 3.09, F = 3.84, *p* = 0.05, η^2^ = 0.04; -Other blame (lower in GDPs): GDPs = 4.32 ± 1.54, HCs = 5.42 ± 1.83, F = 9.10, *p* < 0.01, η^2^ = 0.10.	-Refocusing on planning and putting into perspective appear to indicate gambling-related difficulties, reflected either in more severe symptoms or in more pronounced cognitive distortions.
Bonnaire et al., 2022 [31]	ERQ—French version	10-item ERQ: 6 items for Cognitive Reappraisal, 4 items for Expressive Suppression	GRCS, TAS-20, Emotion Reactivity Scale (ERS), IGT, UPPS-P Impulsive Behavior Scale (short), GDT, Slot Machine Task (SMT)	-Treatment-seeking gamblers had higher expressive suppression (16.46 ± 6.54) than controls (14.46 ± 5.16, *p* = 0.032), and lower cognitive reappraisal (22.81 ± 8.89) than controls (27.07 ± 8.31, *p* = 0.002); -Emotional regulation difficulties, gambling-related cognitions, impulsivity, and behavioural persistence differentiated problem gamblers from non-problem gamblers.	-Gambling persistence (Persistence at the slot machine had the strongest predictive value); -Several emotional (dys)regulation processes (emotional reactivity, expressive suppression and cognitive reappraisal) significantly predicted clinical status.
Ruiz de Lara et al., 2019 [32]	CERQ—Spanish version	9 strategies: Self-blame, Other-blame, Rumination, Catastrophizing, Putting into perspective, Positive refocusing, Positive reappraisal, Acceptance, Refocus on planning	SOGS, GRCS, UPPS-P Impulsive Scale	-Positive urgency predicted control illusion (B = 0.147, *p* = 0.028) and predictive control (B = 0.146, *p* = 0.014). Sensation seeking predicted predictive control (B = 0.173, *p* = 0.004), interpretative bias (B = 0.211, *p* < 0.001), and gambling expectancies (B = 0.266, *p* < 0.001). Reappraisal predicted control illusion (B = 0.173, *p* = 0.006), predictive control (B = 0.112, *p* = 0.043), and interpretative bias (B = 0.147, *p* = 0.010). Blaming others predicted all GRCS dimensions (e.g., control illusion: B = 0.189, *p* = 0.002). Rumination predicted predictive control (B = 0.139, *p* = 0.012); -Gamblers may use relatively sophisticated emotion-regulation strategies, including those typically considered adaptive, while simultaneously exhibiting strong cognitive distortions.	-Gambling seems to be easily triggered by a lack of positive experiences (rather than by the presence of negative ones); -The associations between impulsivity and gambling cognitions were specific to emotion- and motivation-driven facets of impulsivity; -Self-serving emotion regulation strategies were linked to a greater tendency to endorse biased gambling-related beliefs.
Mari et al., 2024 [9]	ERQ— Italian version	10-item self-report; two subscales: Cognitive Reappraisal and Expressive Suppression. Rated on a 7-point Likert scale.	SOGS, Drug Abuse Screening Test (DAST-10), Alcohol Use Disorder Identification Test (AUDIT), Barratt Impulsiveness Scale (BIS-11), TAS-20, Difficulties in Emotion Regulation Scale (DERS-36), PANAS, IGT, GDT, Gambling Affective Task (GAT).	-Risky betting positively correlated with impulsivity, alexithymia, and emotion dysregulation; -Positive affective priming associated with lower bet amounts and longer response times (protective factor); -GD and Substance-Dependent Gambling groups showed higher motor and non-planning impulsivity; -Negative affect significantly higher in GD group; -Risky betting (GAT) correlated with: Risky choices in Game of Dice Task: r = 0.368, *p* < 0.01; Emotion regulation difficulties (DERS strategies): r = 0.435, *p* < 0.01 and Cognitive reappraisal (ERQ): r = −0.429, *p* < 0.01.	-Impulsivity (motor and non-planning); -Negative affect; -Emotion dysregulation; -Low cognitive reappraisal; -Alexithymia.
Mena-Moreno et al., 2022 [33]	ERQ—Spanish version	10-item questionnaire assessing cognitive reappraisal and emotional suppression tendencies	SOGS, Symptom Checklist—Revised (SCL-90-R), DERS, UPPS-P Impulsive Behavior Scale	-Experimental group had fewer relapses, higher compliance, significant reduction in ERQ suppression and general psychopathology.	-Impulsivity; -Emotion regulation; -Emotional suppression; -Psychological distress.
Rogier et al., 2021 [34]	ERQ— Italian version	10-item questionnaire with 2 scores: Cognitive Reappraisal and Expressive Suppression	PGSI (Problem Gambling Severity Index), DES-II (Dissociative Experience Scale, 3 subscales: Depersonalization/Derealization, Amnesia, Absorption)	-Individuals with GD showed lower cognitive reappraisal (27.97 ± 7.10 vs. 30.67 ± 6.31; F = 6.02, *p* = 0.015) and higher expressive suppression (16.53 ± 5.75 vs. 13.80 ± 5.78; F = 6.97, *p* = 0.009) than controls; -Reappraisal was negatively associated with gambling severity (r = −0.35, *p* < 0.001), whereas suppression was positively associated (r = 0.27, *p* = 0.008) and both independently predicted GD severity (β = −0.36 and β = 0.27, respectively).	-Lower Cognitive Reappraisal; -Higher Expressive Suppression; -Higher Dissociation (especially Amnesia and Absorption).

Abbreviations: Gambling Disorder (GD), Emotion Regulation Questionnaire (ERQ), Cognitive Emotion Regulation Questionnaire (CERQ), Difficulties in Emotion Regulation Scale (DERS), South Oaks Gambling Screen (SOGS), Gambling Related-Cognitions Scale (GRCS), Iowa Gambling Task (IGT), Toronto Alexithymia Scale (TAS-20), Game of Dice Task (GDT), Positive and Negative Affect Schedule (PANAS), ), Slot Machine Task (SMT), Emotion Reactivity Scale (ERS), Symptom Check-list—Revised (SCL-90-R), PGSI (Problem Gam-bling Severity Index), DES-II (Dissociative Experience Scale).

## Data Availability

Data are available on request from the corresponding authors.

## Supplementary Materials

The following supporting information can be downloaded at: https://www.mdpi.com/article/10.3390/clinpract16030056/s1, File S1: PRISMA 2020 checklist, File S2: Description of the variables included in the data extraction process.

## Abbreviations

The following abbreviations are used in this manuscript:

ADHD	Attention Deficit Hyperactivity Disorder
AUDIT	Alcohol Use Disorder Identification Test
BIS-11	Barratt Impulsiveness Scale
CBT	Cognitive behavioral therapy
CERQ	Cognitive Emotion Regulation Questionnaire
CES	Centrality of Event Scale
DAST-10	Drug Abuse Screening Test
DERS	Difficulties in Emotion Regulation Scale
DES-II	Dissociative Experience Scale
DSM-5	Diagnostic and Statistical Manual of Mental Disorders, Fifth Edition
ERQ	Emotion Regulation Questionnaire
ERS	Emotion Reactivity Scale
fMRI	Functional Magnetic Resonance Imaging
GAT	Gambling Affective Task
GDT	Game of Dice Task
GD	Gambling Disorder
GRCS	Gambling-Related Cognitions Scale
IGT	Iowa Gambling Task
JBI	Joanna Briggs Institute
OCD	Obsessive–Compulsive Disorder
PANAS	Positive and Negative Affect Schedule
PID-5	Personality Inventory for DSM-5
PGSI	Problem Gambling Severity Index
PRISMA	Preferred Reporting Items for Systematic Reviews and Meta-Analyses
SCL-90-R	Symptom Checklist—Revised
SMT	Slot Machine Task
SOGS	South Oaks Gambling Screen
TAS-20	Toronto Alexithymia Scale
