# Supplementary material for: Assessment of Cognitive Emotion Regulation in Gambling Disorder: A Systematic Review of the Literature
Source: Clin Pract. 2026 Mar 5;16(3):56. doi: 10.3390/clinpract16030056 (PMC13025003; doi:10.3390/clinpract16030056)
Supplement: Supplementary file 1 [file clinpract-16-00056-s001.zip › File S2- Description of the variables included in the data extraction process..pdf]

Description of the variables included in the data extraction process

The following variables were systematically extracted from each included study by two independent reviewers using a structured data extraction form.

Category	Variable	Data to be Extracted
**Study Identification**	First author	
	Year of publication	
	Country of study	
**Methodological Characteristics**	Study design (e.g., cross-sectional, case–control, RCT)	
	Diagnostic criteria for gambling disorder (e.g., DSM-IV, DSM-5, ICD, SOGS)	
	Recruitment setting (clinical / community)	
**Sample Characteristics**	Total sample size	
	Gambling disorder group size	
	Control group size (if applicable)	
	Sex distribution (M/F)	
	Mean age ± SD	
	Inclusion criteria	
	Exclusion criteria	
**Gambling Characteristics**	Type of gambling activity (e.g., slot machines, sports betting, mixed)	
**Emotion Regulation Measures**	Instrument used (ERQ / CERQ)	
	Language/version of the scale	
	Scale version description (e.g., number of items, subscales: cognitive reappraisal, expressive suppression; CERQ strategies)	
**Additional Psychological Measures**	Impulsivity measures (e.g., UPPS-P, BIS-11)	
	Emotion dysregulation measures (e.g., DERS)	
	Cognitive distortions measures (e.g., GRCS)	
	Alexithymia measures (e.g., TAS-20)	
	Gambling severity measures (e.g., SOGS, PGSI)	
	Other scales applied	
	Neuropsychological / neuroimaging measures (if applicable)	
**Key Outcomes and Findings**	Main GD vs. control group differences	
	Associations between emotion regulation strategies and gambling severity	
	Identified predictors of gambling disorder severity	
	Study limitations	
